# Are scurs in heterozygous polled (*Pp*) cattle a complex quantitative trait?

**DOI:** 10.1186/s12711-020-0525-z

**Published:** 2020-02-07

**Authors:** Lilian Johanna Gehrke, Aurélien Capitan, Carsten Scheper, Sven König, Maulik Upadhyay, Kristin Heidrich, Ingolf Russ, Doris Seichter, Jens Tetens, Ivica Medugorac, Georg Thaller

**Affiliations:** 1 Institute of Animal Breeding and Husbandry, Christian-Albrechts-University Kiel, 24098 Kiel, Germany; 2 Vereinigte Informationssysteme Tierhaltung w.V. (Vit) Verden, 27283 Verden, Germany; 3 Université Paris-Saclay, INRAE, AgroParisTech, GABI, 78350 Jouy-en-Josas, France; 4 Institute of Animal Breeding and Genetics, Justus-Liebig-University of Gießen, 35390 Gießen, Germany; 5 Population Genomics Group, Department of Veterinary Sciences, Ludwig Maximillians University Munich, Munich, Germany; 6 Tierzuchtforschung e.V. München, Grub, Germany; 7 Department of Animal Sciences, Georg-August University, 37077 Göttingen, Germany; 8 Center for Integrated Breeding Research, Georg-August-University, 37077 Göttingen, Germany

## Abstract

**Background:**

Breeding genetically hornless, i.e. polled, cattle provides an animal welfare-friendly and non-invasive alternative to the dehorning of calves. However, the molecular regulation of the development of horns in cattle is still poorly understood. Studying genetic characters such as polledness and scurs, can provide valuable insights into this process. Scurs are hornlike formations that occur occasionally in a wide variety of sizes and forms as an unexpected phenotype when breeding polled cattle.

**Methods:**

We present a unique dataset of 885 Holstein–Friesian cattle with polled parentage. The horn phenotype was carefully examined, and the phenotypic heterogeneity of the trait is described. Using a direct gene test for polledness, the polled genotype of the animals was determined. Subsequently, the existence of a putative *scurs* locus was investigated using high-density genotype data of a selected subset of 232 animals and two mapping approaches: mixed linear model-based association analyses and combined linkage disequilibrium and linkage analysis.

**Results:**

The results of an exploratory data analysis indicated that the expression of scurs depends on age at phenotyping, sex and polled genotype. Scurs were more prevalent in males than in females. Moreover, homozygous polled animals did not express any pronounced scurs and we found that the Friesian *polled* allele suppresses the development of scurs more efficiently than the Celtic *polled* allele. Combined linkage and linkage disequilibrium mapping revealed four genome-wide significant loci that affect the development of scurs, one on BTA5 and three on BTA12. Moreover, suggestive associations were detected on BTA16, 18 and 23. The mixed linear model-based association analysis supports the results of the combined linkage and linkage disequilibrium analysis. None of the mapping approaches provided convincing evidence for a monogenic inheritance of scurs.

**Conclusions:**

Our results contradict the initial and still broadly accepted model for the inheritance of horns and scurs. We hypothesise an oligogenetic model to explain the development of scurs and polledness.

## Background

Horns are a characteristic and variable trait in cattle and their main role is self-defence in wild life. In the past, horns of domesticated cattle were used for tethering and attachment to harnesses [1]. However, in modern cattle industry, hornless cattle are desired for practical and economic reasons, such as reduced risk of injuries for humans and conspecifics and easier handling of the animals. To date, 80% of dairy, 46% of beef, and 67% of suckler calves in Europe are dehorned or disbudded [2]. Public animal welfare stakeholders have criticized this routinely performed dehorning of calves and raised awareness of the agricultural industry. Breeding genetically hornless, i.e. polled cattle, provides a long-term solution to these issues.

Since the rediscovery of Mendel’s laws of heredity [3, 4], many studies on the inheritance of horns have been conducted, and rapidly, polledness was described as an autosomal dominant trait. These studies also reported the unexpected occurrence of scurs in polled cattle. Scurs are described as hornlike formations that grow in the same area as horns and are only loosely attached to the skull [5, 6]. It is not clear at what age scurs develop, but they are assumed to occur later in life than horns [7]. The occurrence of scurs hampers the advantages that could be achieved by breeding for polledness as they bear an injury risk and thus make dehorning necessary again. In addition, they could be perceived as an anomaly leading to an uncertain inheritance of polledness and, in practice, decrease the acceptance of diffident farmers.

The development of horns results from the interaction between tissues that originate from the ectoderm and mesoderm and from their transformation, and seems to be programmed during embryogenesis [8], most likely at 60 days of gestation [9]. It is generally acknowledged, that the bony core of the horn develops from a separate ossification centre and fuses with the skull afterwards [7, 10]. However, the whole mechanism of the development of horns is not yet completely understood. The characterization of the genetic basis of polledness and scurs can contribute to a better understanding of the molecular mechanisms that influence the development of horns. Moreover, it can provide valuable knowledge about how different tissues and cell differentiation work together during organogenesis [11–13].

The *polled* locus was mapped to the proximal end of bovine chromosome 1 (BTA1) [14–16] and four variants were identified (OMIA 000483-9913); among these, two i.e. the Friesian (*P*_*F*_) and Celtic (*P*_*C*_) variants, are common in European cattle breeds [1, 17–19] and enable direct selection for polledness and the set-up of appropriate breeding strategies.

With respect to scurs, the most commonly accepted model of inheritance was initially proposed by White and Ibsen [6] and revised by later studies [6, 20, 21]. The model assumes that four biallelic loci interact to control the development of horns: the “symbolic” *horn* locus (*H*, which is suggested to be always present and homozygous), the *polled* locus (with alleles *P* for polled and *p* for horns), the *scurs* locus (*Sc* for scurs and *sc* for no scurs) and the *African horn* locus (*Ha* for African horns and *ha* for no African horns) [6, 20]. Scurs are expected to be masked in otherwise horned animals (*p*/*p*), but there is discussion about an epistatic interaction between the *polled* and *scurs* loci. However, in the literature contradictory results are reported on the mapping and expression of scurs, and some studies discuss whether the development of scurs depends on sex, the genotype at the *polled* locus, and heterogeneity at the *scurs* locus [7, 14, 20, 22, 23]. Interestingly, Capitan et al. [24] identified a phenotype similar to scurs, called type 2 scurs, which is caused by a mutation within the *TWIST1* gene that occurred independently from the polled genotype and is limited to a single Charolais family.

In this study, we present a unique dataset of 885 Holstein–Friesian cattle with a precisely examined horn phenotype. For the first time, we describe the diversity of the scurs phenotype in Holstein–Friesian cattle in detail. We present new insights into the inheritance pattern and expression of scurs and an exhaustive mapping study of a putative *scurs* locus using two mapping approaches.

## Methods

### Animals and phenotyping

**Fig. 1:**
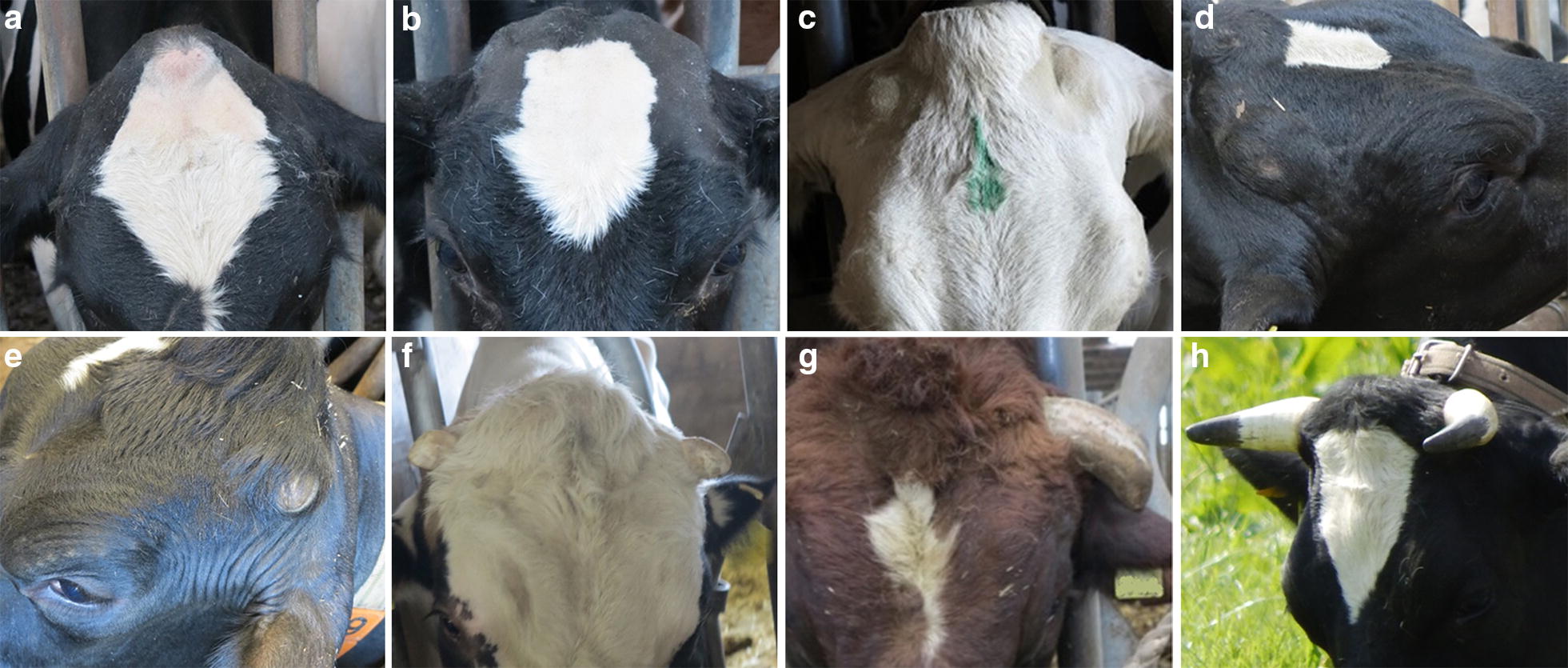
Observed horn phenotypes. **a** Smoothly polled. **b** Small frontal bumps. **c** Frontal bumps. **d–g** Small to long scurs. **h** Horns

In this study, our aim was to investigate the scurs phenotype, its genetic architecture and inheritance pattern, and a putative *scurs* locus in Holstein–Friesian cattle. We surveyed 885 Holstein–Friesian cattle housed on German dairy cattle farms that are actively breeding for polledness and do not routinely dehorn calves. To obtain an adequate number of male phenotypes, we surveyed the bulls of three German breeding companies. Only individuals that descended from at least one polled parent were investigated. In total, we phenotyped 885 (813 females and 72 males) Holstein–Friesian cattle that were between 1 to 133 months old and housed on 20 farms. All individuals were examined by the same person, who inspected and palpated the left and right horn area. If necessary, the horn area was shaved to screen for small scabs or scars. Observed phenotypes were classified into five categories: (i) “smoothly polled”: absence of horns or any corneous growth in the horn area; (ii) “small frontal bumps”: small bulges in the horn area that are probably due to ossification; (iii) “frontal bumps”: pronounced bulges in the horn area (osseous base with a rather thick tissue layer) and no keratinization of the skin; (iv) “scurs”: hornlike formations in the horn area that are loosely attached to the skull by soft tissue and that vary from frontal bumps with a keratinization of the covering skin to long hornlike formations (up to 15 cm); and (v) “horns”: regular horns that are firmly attached to the skull (see Fig. 1). Individuals with an intermediate (i.e. laterally diverging) phenotype were classified as “others”, e.g. a smoothly polled left horn area and a right horn area with a small scur.

### Coding of phenotypes for mapping

In order to test the characteristics of the phenotype in the mapping approaches, horn phenotypes were converted into codings (Table 1). We applied two continuous (CC and CCL) and two binary (BC1 and BC2) coding models of phenotypes. In model CC, horn status was coded as a linear type trait with five classes and in model CCL, it was transformed on a liability scale with N(0,1) according to the frequency of occurrence (see Additional file 1: Figure S1). In models BC1 and BC2, horn status was converted into a binary trait with bumps counted as controls or cases. These codings of the horn phenotype are listed in Table 1.

**Table 1:** Coding of observed phenotype categories for 232 high-density genotyped animals with four models

Coding	Smoothly polled (n = 75)	Frontal bumps (n = 130)	Scabs (n = 19)	Small scurs (0.5–4.99 cm) (n = 7)	Medium scurs (5–10 cm) (n = 1)
CC	1	2	3	4	5
CCL	− 1.111	0.292	1.458	2.110	2.939
BC1	1	2	2	2	2
BC2	1	1	2	2	2

### Genotypes

DNA was extracted from whole blood or hair samples by applying a modified protocol according to Miller et al. [25]. To determine the polled genotype, a direct gene test was performed on all 885 animals [1, 17], which allows differentiation between the Friesian (*P*_*F*_) and the Celtic *polled* (*P*_*C*_) variants.

For single nucleotide polymorphism (SNP) genotyping, we selected a subset of animals based on the following criteria: (i) horn phenotype categories “smoothly polled”, “frontal bumps” and “scurs”; (ii) minimum age of 700 days for animals of the categories “smoothly polled” and “frontal bumps”; (iii) female animals; and (iv) heterozygous polled animals that carry the *P*_*F*_ variant. The selected subset consisted of 240 cows, which were genotyped with the BovineHD BeadChip (Illumina, Chicago) that contains 777,962 SNPs. SNPs were remapped to the bovine reference genome assembly ARS-UCD1.2 [26]. During quality control, SNPs with an unknown position, a minor allele frequency (MAF) lower than 0.01 and a call rate per marker lower than 0.9 were excluded. Moreover, all genotyped X chromosome SNPs were divided into pseudo-autosomal XY SNPs and X chromosome-specific SNPs. After quality control, 762,966 SNPs and 232 individuals with a genotype call rate higher than 0.95 remained for analysis. We imputed missing genotypes and reconstructed haplotypes using the software BEAGLE 5 [27, 28], which exploits haplotype Hidden Markov models. BEAGLE 5 considers the relationships between genotyped individuals nested in a linkage-format pedigree. For a better accuracy, genotype and pedigree information of 1434 additional animals, which were genotyped with the BovineHD BeadChip (call rate > 0.95) and otherwise not included in the following mapping, were added for haplotyping and imputation.

### Genetic parameters

We estimated the phenotypic variance explained by all SNPs by using the GCTA software version 1.92.3 and applying a genomic-relatedness-based restricted maximum-likelihood (GREML) approach [29]. The age at phenotyping was corrected by including it as a covariate in the model. The analysis was performed for all phenotype codings. As validation of the results, for each phenotype coding, 100 permutations of each phenotype were conducted and the heritability was estimated.

### Mapping approaches

#### Association analysis based on a mixed linear model

To map a putative *scurs* locus, we conducted mixed linear model based association analyses (MLMA) with a leave-one-chromosome-out (LOCO) approach as implemented in the GCTA software version 1.92.3 [29]. The following model was applied:

$${{\mathbf{y}} = \mathbf{Xb} + \mathbf{Z}}_{\mathbf{a}} {\mathbf{a} + \mathbf{Z}}_{\mathbf{u}} {\mathbf{u} + \mathbf{e},}$$

where ${\mathbf{y}}$ is the vector of horn phenotypes, ${\mathbf{b}}$ is the vector of fixed effects including the overall mean and age at phenotyping, ${\mathbf{a}}$ is the vector of the additive effect (fixed) of the SNP tested for association, ${\mathbf{u}}$ is the vector of the accumulated effects (random) of all SNPs excluding those on the chromosome that carries a candidate SNP, ${\mathbf{e}}$ is the vector of residuals. ${\mathbf{X}}$, ${\mathbf{Z}}_{{\mathbf{a}}}$ and ${\mathbf{Z}}_{{\mathbf{u}}}$ are the incidence matrices for ${\mathbf{b}}$, ${\mathbf{a}}$ and ${\mathbf{u}}$, respectively. Based on the Bonferroni method, significance thresholds were set to a genome-wide significance at P < 0.05/N and a suggestive significance at P < 1/N, where N is the number of SNPs used in the analysis [30]. The genome-wide significance threshold in this study was equal to 6.55 × 10^− 8^ (0.05/762966) and the suggestive significance threshold to 1.31 × 10^− 6^ (1/762966).

#### Combined linkage disequilibrium and linkage analysis

Another approach to map a putative *scurs* locus used a combined linkage disequilibrium and linkage analysis (cLDLA), which is the method proposed by Meuwissen et al. [31]. To correct for population stratification and family relationships, a unified additive relationship matrix (${\mathbf{G}}$) between all animals and its inverse (${\mathbf{G}}^{ - 1}$) were estimated [32]. We also implemented the LOCO approach. A chromosome with n SNPs has n–1 SNP intervals and their corresponding midpoints. Each SNP interval midpoint was considered as a putative locus with a causal effect on the investigated phenotype. We used the surrounding SNP haplotypes (reconstructed as described above) and a sliding window (sw) approach to estimate the identity-by-descent (IBD) between alleles at each SNP interval midpoint along the chromosomes. Thus, the SNP window shifts SNP by SNP along the chromosome, e.g. a sliding window with 40 SNPs (sw40) overlaps by 39 sequential SNPs. Different window sizes of 20, 40, 80 and 160 sequential SNPs were tested. For each window midpoint, e.g. for sw40 between SNPs 20 and 21, a locus IBD matrix was estimated as described by Meuwissen and Goddard [33]. Then, the locus IBD matrix was converted into a diplotype relationship matrix (D_RM_) as suggested by Lee and van der Werf [34].

The genome-wide QTL mapping was performed using a cLDLA approach as described in Medugorac et al. [18]. In the mixed linear model, linkage disequilibrium was considered in the D_RM_, whereas linkage was accounted for in the reconstruction of haplotypes. Variance component analysis for each window midpoint was carried out with ASReml [35]. ASReml estimates the maximum likelihood, variance components, and fixed and random effects simultaneously by taking the genome-wide additive relationships (${\mathbf{G}}$) as well as the IBD probabilities of the putative causal locus (i.e. QTL) into account. The following mixed linear model was applied:

$${\mathbf{y}} = {\mathbf{X}}{\varvec{\upbeta}} + {\mathbf{Z}}_{1} {\mathbf{u}} + {\mathbf{Z}}_{2} {\mathbf{q}} + {\mathbf{e}}$$

where ${\mathbf{y}}$ is the vector of the alternative horn phenotype codings converted into a binary or quantitative trait; ${\varvec{\upbeta}}$ is the vector of fixed effects including the overall mean ($\mu$) and age at phenotyping; ${\mathbf{u}}$ is the vector of n random polygenic effects for each animal with ${\mathbf{u}}\sim {\text{N}}\left( {0,{\mathbf{G}}\sigma_{{\mathbf{u}}}^{2} } \right)$; ${\mathbf{q}}$ is the vector of random additive genetic effects of the putative QTL with ${\mathbf{q}}\sim {\text{N}}\,\,\left( {0,\,\,{\mathbf{D}}_{{{\mathbf{RM}}_{i}}} \,\sigma_{\text{q}}^{2} } \right)$ , where ${\mathbf{D}}_{{{\mathbf{RM}}_{i} }}$ is the D_RM_ matrix at the $i\text{th}$ marker interval midpoint along the chromosome. Random residual effects were included in the vector ${\mathbf{e}}$ with ${{\mathbf{e}\sim }\,\text{N}\left( {{0,{\mathbf{I}}\sigma }_{\text{e}}^{\text{2}} } \right)}$ , where ${\mathbf{I}}$ is an identity matrix. Random effects (${\mathbf{u}}$, ${\mathbf{q}}$, ${\mathbf{e}}$) are assumed to be uncorrelated and normally distributed. Their respective variances ($\sigma_{{\mathbf{u}}}^{2}$, $\sigma_{{\mathbf{q}}}^{2}$, and $\sigma_{{\mathbf{e}}}^{2}$) were estimated simultaneously using ASReml. The matrices ${\mathbf{X}}$, ${\mathbf{Z}}_{1}$ and ${\mathbf{Z}}_{2}$ are the incidence matrices for the fixed and random effects.

Finally, a likelihood ratio test (LRT) for the goodness-of-fit between the null hypothesis ($H_{0}$: model without a QTL effect) and the alternative hypothesis ($H_{1}$: model including a QTL effect) at each SNP interval midpoint was calculated. The logarithms of likelihood estimated by ASReml were compared as follows:

$$LRT = - 2*\left( {logL\left( {H_{0} } \right) - logL\left( {H_{1} } \right)} \right)$$

To empirically estimate the genome-wide significance thresholds, we conducted a cLDLA permutation in the investigated mapping population. For each chromosome, 100 datasets with randomized phenotypes were tested at 100 random SNP interval midpoints each. This resulted in 10,000 LRT values per chromosome (29 autosomes, X and pseudoautosomal XY), i.e. 310,000 randomized LRT values genome-wide. The 15 highest randomized LRT values defined the genome-wide threshold of falsely rejecting the null hypothesis at α = 0.0005 (15/310,000) for a particular design, i.e. phenotype coding and window size. Due to computation time limits, the permutation test was conducted for the CC coding for all window sizes, and for all other codings for sw40 only.

#### Power calculations

To estimate the power of the design, a simplified simulation study was conducted. Briefly, liabilities for 232 independent animals were generated on the scale N(0,1). Individual liabilities were the sum of the QTL effect and of independent residuals on the underlying scale, where the QTL explained 10, 20 and 30% of the genetic variation assuming a heritability of 0.6, respectively. Liabilities were then converted into the respective codings as defined in Table 1 (CC, CCL, BC1) according to appropriate thresholds, which ensure the real distribution of phenotypes. Finally, a single marker regression of r^2^ = 0.8 for a SNP in linkage disequilibrium with the QTL was conducted and the *P* value of the regression coefficient was compared to the suggestive and genome-wide significance thresholds defined above. Each scenario (QTL-variance*coding) was repeated 10,000 times and the proportion of replicates with P-values exceeding the significance thresholds represents its power.

### Annotation of gene content and gene set enrichment analysis

Annotation of gene content was performed as described by Medugorac et al. [18]. Briefly, the genes in 200-kb intervals surrounding the significant regions that were detected with the CC and CCL phenotype coding were extracted from the UCSC Genome Browser (ARS-UCD1.2) [26]. For intervals without genes, we considered the gene that reads in the 5′ to 3′ sense and was closest to the detected region within a 1-Mbp surrounding interval. We used the “RefSeq Genes” track, as well as the “Non-cow RefSeq genes”, “Cow mRNAs from GenBank” and “Cow ESTs that have been spliced” tracks to consider genes that might have been missed in the annotation of the bovine genome assembly ARS-UCD1.2. Only the genes that have been annotated in the human or mouse genome were considered. Gene set enrichment analysis for MGI Mammalian Phenotype Level 4 2019 (MMP4) was performed with Enrichr [36–38].

## Results

### Phenotyping and polled genotype

**Fig. 2:**
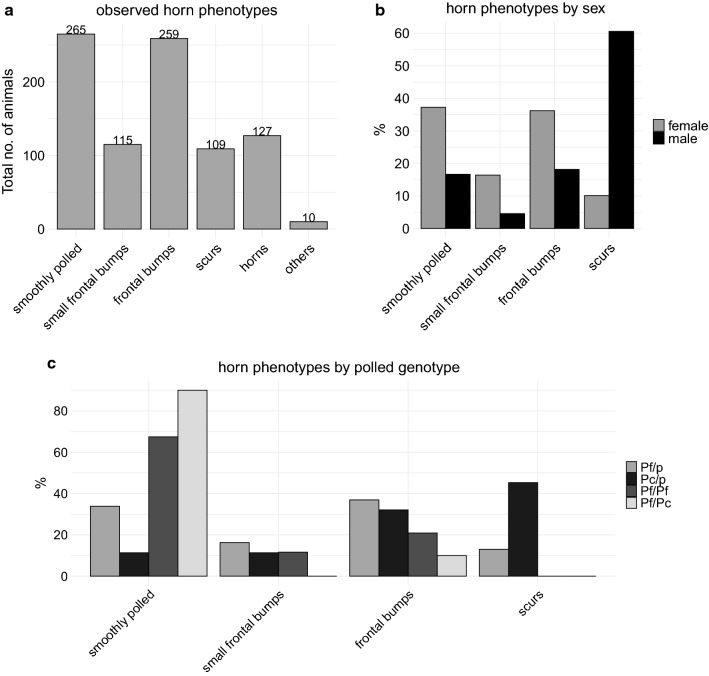
Distributions of horn phenotypes. **a** Total number of animals in the horn phenotype categories: smoothly polled, small frontal bumps, frontal bumps and scurs. **b** Proportion of male and female cattle in the horn phenotype categories: smoothly polled, small frontal bumps, frontal bumps and scurs, with number of males = 66 and number of females = 683. **c** Proportion of heterozygous polled animals carrying the Friesian (*P*_*F*_/*p*, n = 615) or the Celtic (*P*_*C*_/*p*, n = 53) polled variant and homozygous polled animals carrying the Friesian polled variant in homozygous state (*P*_*F*_/*P*_*F*_, n = 43) and carrying one Friesian and one Celtic polled variant (*P*_*F*_/*P*_*C*_, n = 10) in the different horn phenotype categories (smoothly polled, small frontal bumps, frontal bumps and scurs)

Among the 885 surveyed Holstein–Friesian cattle, we observed 265 smoothly-polled animals, 115 individuals with small frontal bumps and 259 with frontal bumps, 109 animals showed scurs, and 127 animals were horned. For the remaining 10 individuals, we were not able to unambiguously state the horn phenotype and these were classified as others (see Fig. 2a). It should be mentioned that since we focused on animals that descended from at least one polled parent, the number of horned animals was small. In the following analysis, we excluded animals that were classified in the ‘horn’ and ‘others’ categories, i.e. 137 animals, since they were not expected to contribute any additional relevant information to our study.

To investigate whether the expression of scurs is independent from sex, we compared the proportion of male and female individuals in the different horn phenotype categories. Strikingly, relatively more male (60%) than female individuals (10%) showed scurs, whereas more females (37% vs. 17%) were smoothly-polled or had small frontal bumps (17% vs. 5%) and frontal bumps (36% vs. 18%) (Fig. 2b). A Pearson’s Chi squared test confirmed that the horn phenotype is not independent from sex (p < 2.2 × 10^− 16^).

Furthermore, we investigated, whether the expression of scurs is independent from the polled genotype. A direct gene test for polledness allowed us to distinguish between the *P*_*F*_ and *P*_*C*_ variants. None of the homozygous polled (*P*_*F*_*/P*_*F*_ or *P*_*F*_*/P*_*C*_) animals had scabs or scurs, but most of them were smoothly-polled (Fig. 2c); only 15 of the 53 homozygous polled animals showed small frontal bumps (*P*_*F*_*/P*_*F*_, n = 5) or frontal bumps (both *P*_*F*_*/P*_*C*_, n = 1 and *P*_*F*_*/P*_*F*_, n = 9; Fig. 2c). It is also striking that heterozygous animals that carry the *P*_*C*_ allele expressed a significantly higher proportion of scurs than the heterozygous animals that carry the *P*_*F*_ allele; this was confirmed by a Fisher’s exact test (P < 3.275 × 10^− 7^).

### Genetic parameters and mapping analysis of the *scurs* locus

To map the putative *scurs* locus, we genotyped a subset of selected female animals on the BovineHD BeadChip from Illumina. The phenotypic variance explained by all SNPs was estimated using the GREML approach. For all phenotype codings, SNP heritability was estimated for the observed phenotype and for 100 permutations of this phenotype. The estimated phenotypic variance explained by all SNPs was 0.65 (± 0.19) for the CC coding, 0.63 (± 0.20) for CCL, 0.60 (± 0.25) for BC1, and 0.41 (± 0.18) for BC2. All estimates of the SNP-based heritability of the phenotype differed significantly from those of the permuted phenotype. The detailed results on the estimates for the different codings and respective permutations are in Additional file 2: Table S1. The estimated SNP heritability was much lower for the BC2 coding than for all the other codings and thus, was not analysed further.

**Fig. 3:**
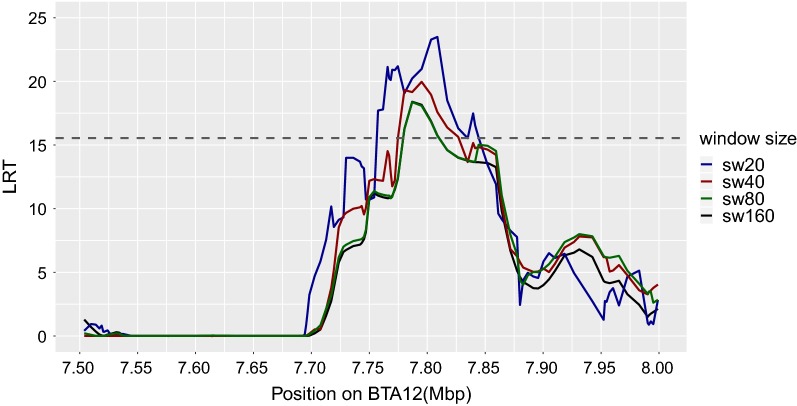
Comparison of LRT values from the cLDLA analysis with different window sizes. LRT results of sliding windows (sw) of 20, 40, 80 and 160 subsequent SNPs for a detected region on BTA12

Different window sizes, i.e. sw20, sw40, sw80, and sw160, for cLDLA were tested. The LRT curves became smoother and less peaked with increased window size but the mapping results remained similar. Figure 3 is a good example of the influence of window size on the shape of the LRT curve in the QTL region on BTA12. As window size increased, the computation time necessary to calculate the IBD matrices increased significantly. For the mapping population investigated here, the best compromise between LRT curve resilience and acceptable computation time was obtained with sw40. To estimate the empirical genome-wide significance threshold for the cLDLA with different phenotype codings (Table 1), a permutation test for each phenotype coding and for different window sizes was conducted. We detected very similar genome-wide significance thresholds (α = 0.00005; false positive rate of 15/310,000) for sw40 and different codings, i.e. 14.74 for CC, 15.28 for CCL and 14.98 for BC1. A detailed list of the detected thresholds for all codings is in Additional file 2: Table S2.

**Fig. 4:**
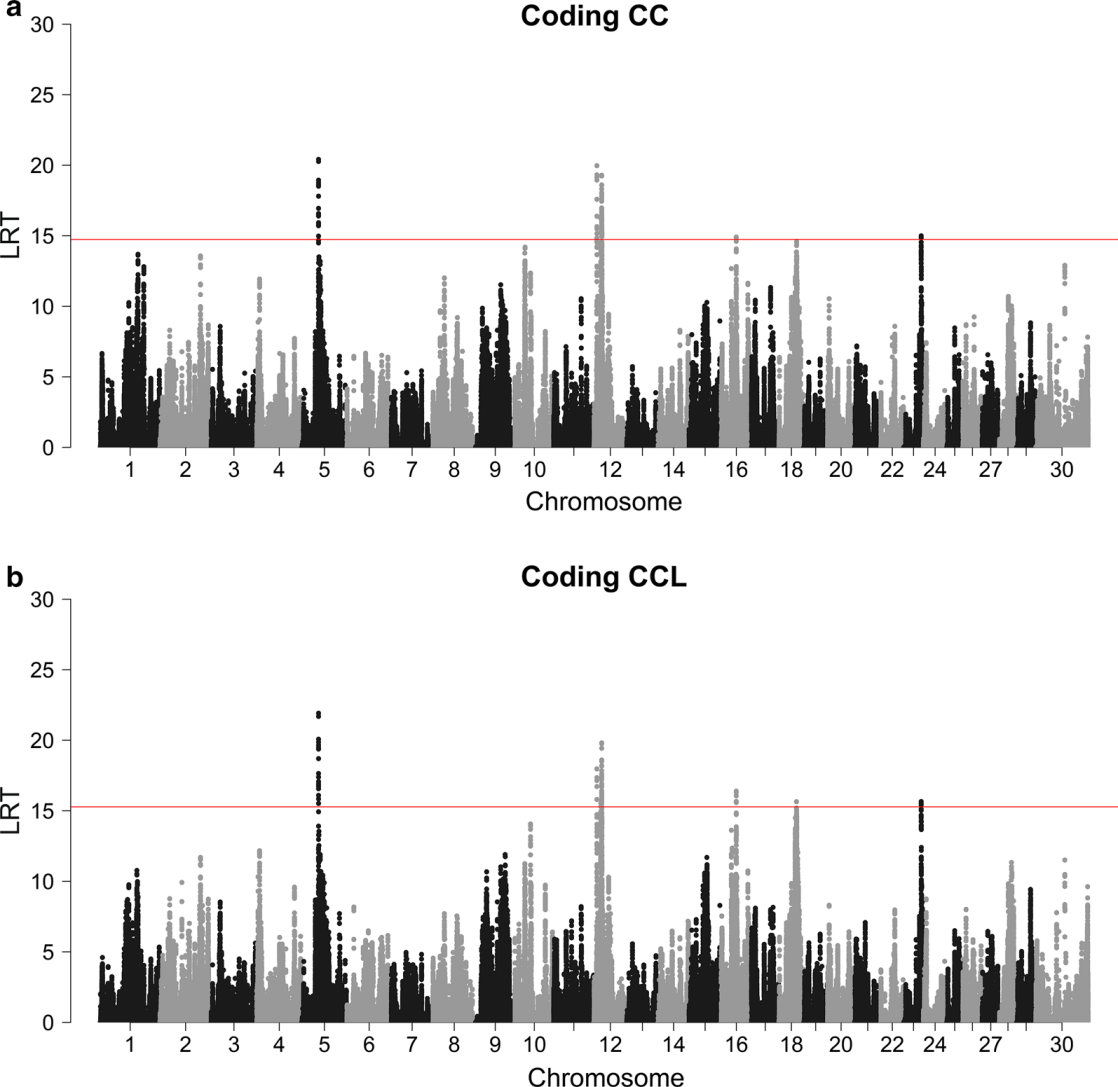
Results of the cLDLA for scurs with different phenotype codings with sw40. LRT- values are shown on the y-axis, bovine chromosomes on the x-axis. The red horizontal line marks the genome-wide significance threshold (α = 0.00005) derived from permutation testing. **a** CC phenotype coding and **b** CCL phenotype coding

The results of the cLDLA (sw40) are shown in the Manhattan plots of Fig. 4 for the two continuous phenotype codings and in Additional file 3: Figure S2 for BC1. With the CC and CCL codings, four genome-wide significant associations were detected. On BTA5, LRT values in the region between 44,657,092 and 44,691,633 bp (i.e. 18 overlapping windows) with the CC coding and between 44,653,747 and 44,695,065 bp (i.e. 20 overlapping windows) with the CCL coding exceeded the significance threshold of 14.74 and 15.28, respectively. On BTA12, three genome-wide significant peaks were found with a continuous coded trait: one peak between 7774,577 and 7844,252 bp (CC) and 7780,281 and 7808,705 bp (CCL); a second peak between 18,556,088 and 18,561,582 bp (CC) and 18,561,582 and 18,609,141 bp (CCL); and a third peak that spanned a larger region between 20,468,696 and 21,192,686 bp (CC) and 20,454,904 and 20,857,664 bp (CCL). These four QTL mapped at genome-wide significance irrespective of the window size (20–160 SNPs) used and of the continuous coding of horn status. Moreover, with different window sizes, the peaks on BTA16, 18 and 23 were close to or just reached the genome-wide significance thresholds. Positions with significant LRT values obtained with the CC and CCL codings and their overlaps are listed in Additional file 4: Table S5. The results obtained with the BC1 coding differed completely from those with both CC and CCL codings (see Additional file 3: Figure S2). The binary coded trait was characterised by a large number of high LRT values that were mostly associated with single SNP windows.

**Fig. 5:**
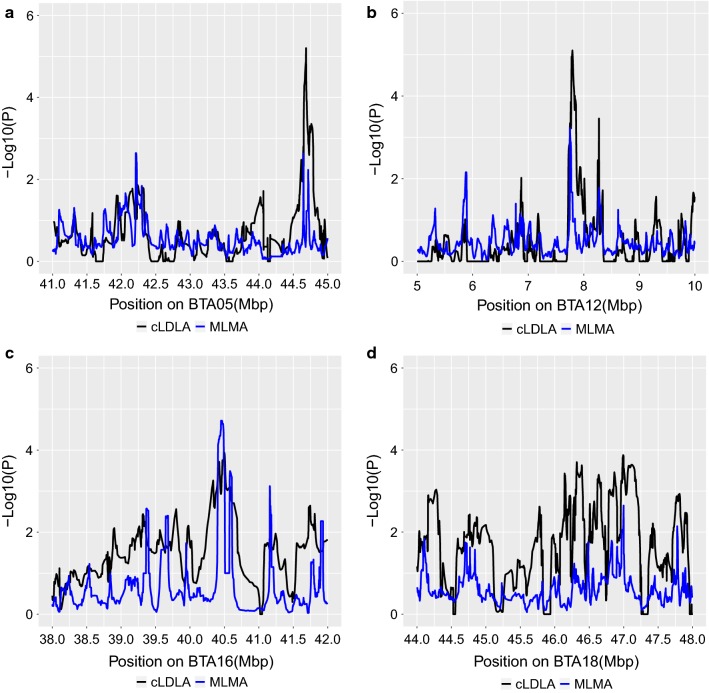
Congruence of cLDLA (sw40) and MLMA results for CC phenotype coding. P-values of both methods were transformed to − log10(P); **a** detected region on BTA5 (41–45 Mb); **b** detected region on BTA12 (5–10 Mb); **c** detected region on BTA16 (38–42 Mb); and **d** detected region on BTA18 (44–48 Mb)

To check the congruence of the cLDLA and GWAS mapping results, we performed a mixed linear model-based association analysis (MLMA) with the procedure implemented in the software GCTA-LOCO [29]. MLMA mapping was performed for CC and CCL coding but for BC1 the analysis was difficult due to convergence problems. To facilitate the visual comparability of the cLDLA and MLMA mapping results, we transformed the P-values from both methods to − log10(P). Thus, we consider that the LRT values follow a χ^2^ distribution with one degree of freedom [39]. It should be kept in mind that MLMA estimates the P-value directly at a specific SNP, whereas cLDLA estimates it at the midpoint between two adjacent SNPs. To interpolate the midpoint values and smooth the curve, we calculated the average of 10 adjacent MLMA − log10(P)-values, which are shown in parallel to the cLDLA values. Figure 5 illustrates the congruence between both mapping methods for significant and indicative QTL on BTA5, 12, 16 and 18 according to CC phenotype coding and sw40.

## Discussion

Close inspection and palpation of 885 Holstein–Friesian skulls revealed a great variety of horn phenotypes, which ranged from smoothly polled animals, through to frontal bumps, scabs, up to 10 cm long scurs, and finally to normal horns. A large proportion (42%) of the animals in our dataset had small frontal bumps or frontal bumps and, according to reports from experienced staff of German breeding associations, the majority of scabs and scurs arise on previously developed frontal bumps. Therefore, we assumed that a frontal bump is a precursor of a scur. However, due to premature culling or unknown environmental factors, some frontal bumps will never develop into scurs.

Since the first study that reported the development of scurs [40], several authors have described scurs and horn phenotypes in various cattle breeds and have sorted them into partly ambiguous categories. For example, in 1952, Williams and Williams [41] divided the observed phenotypes into six categories, although they mention that “the involved phenotypes form an almost continuous series”. In addition to the phenotypes that we have described here, two other phenotypes have been mentioned in the literature: (i) scurs that are partially fused with the frontal bone and therefore rather firmly attached to the skull have been reported in the Charolais and Angus breeds [7, 14], and (ii) mutilated horns that are described as crumpled horns occurring only in females have been found in polled Hereford cattle [41]. Since our data concern Holstein–Friesian cattle, these two phenotypes were not observed in our study.

The currently accepted model for the inheritance of horns and scurs, which is described in the introduction and summarised in the OMIA database (OMIA 000483-9913), was proposed before DNA was even identified as the carrier of genetic information i.e. before it was possible to accurately determine the underlying polled genotypes. This explains that, to date, several inconsistencies with this model have been reported [7, 22, 23]. In addition, by using a larger dataset, we confirm the findings of recent studies, which suggest that homozygous polled animals may never have fully developed scurs [22, 23]. However, we did observe small frontal bumps or frontal bumps, which we assume to be precursors of scurs, in 28% of the homozygous polled animals. Moreover, the phenotypic distribution of our data supports the hypothesis that scurs is a sex-dependent trait, since scurs are significantly more prevalent in *P/p* males than in *P/p* females (61% vs. 10%) and the scurs are on average larger in males than in females. Scurs seem to develop later in life than horns [7] and it is not clear if they occur later in females than in males. However, in our data, the youngest individuals with scurs were six months old and belonged to both sexes.

The segregation of the *P*_*C*_ and *P*_*F*_ alleles in 885 Holstein–Friesian cattle provide further insight into the possible interaction between the polled allele and the development of scurs. According to our dataset, the *P*_*F*_ allele supresses the development of scurs more efficiently (P < 3.27×10^− 7^ ) than the *P*_*C*_ allele.

Phenotypes that are routinely recorded by breeding associations are easily available but, during the pilot project, we noted that some of the animals recorded as smoothly polled at breeding approval developed regular scurs with advancing age. Moreover, such routine phenotyping by several investigators may suffer from lack of standardisation. This emphasises the need for standardised phenotyping and recording of age at phenotyping. The age-dependent penetrance is a well-known problem in mapping studies for genetic traits that are not expressed at birth but develop only later in life [42, 43]. Moreover, frontal bumps can go unnoticed and such misclassification during phenotyping may be another reason why there are few studies on the genetic architecture of scurs and horns [41]. Finally, the direct gene test for polledness has become available only recently and, contrary to our study, precise polled genotypes were not available in most previous ones.

To prove the consistency of the cLDLA results with different window sizes, we performed genome-wide mapping with windows consisting of 20, 40, 80 and 160 SNPs, which allowed us to recommend the best window size as a compromise between LRT curve resilience and reasonable computation time for the estimation of the locus IBD matrices and for variance analyses including ${\mathbf{G}}^{ - 1}$ of constant size and ${\mathbf{D}}_{{{\mathbf{RM}}i}}^{ - 1}$ of variable size for position ($i$) along the genome. For genome-wide mapping with a window size of 40 SNPs, we estimated 653,668 ${\mathbf{D}}_{{{\text{RM}}_{i} }}$ matrices, inverted them, and performed the same number of variance analyses by ASReml [35]. As window size increased, the number of matrices and variance analyses remained the same, but the size of the matrices increased. The computing expense is an exponential function of the matrix size, which depends on the length of the haplotype considered (window size) and on the haplotype diversity in the mapping population. Our results suggest that a genome-wide QTL scan with a shorter window (e.g. 20 SNPs) and subsequent confirmation of significant and indicative QTL with a longer window could be a good compromise, especially for larger mapping populations.

According to the collected data and previously published results [7, 22, 24, 44], the genetic basis of the scurs phenotype is complex and affected by the polled genotype including allelic heterogeneity (*P*_*C*_*/P*_*C*_, *P*_*F*_*/P*_*F*_, *P*_*C*_*/P*_*F*_, *P*_*C*_*/p*, *P*_*F*_*/p*) as well as sex and age of the individuals at phenotyping. However, by considering the initial hypothesis that scurs is a monogenic qualitative trait, we performed binary coding of the horn status (BC1). To exclude any source of noise as much as possible, we analysed only the heterozygous *P*_*F*_*/p* females. As discussed above, we consider frontal bumps as precursors of scurs, and thus, the first binary coding (BC1) puts frontal bumps, scabs and scurs into the same class. However, there is no keratin layer on the bulges in the horn area in the case of animals classified as “small frontal bumps” or “frontal bumps”. Thus, the second binary coding (BC2) puts “smoothly polled”, “small frontal bumps” and “frontal bumps” in the first class, and animals with keratinization of the skin in the horn area (from “scabs” to large “scurs”) into the second class. These two binary traits (BC1 and BC2) and two continuous coded traits (CC and CCL) were analysed. Estimates of the SNP heritability were rather high for all phenotype codings (see Additional file 2: Table S1). Estimation of the phenotypic variance explained by all SNPs depends on the underlying dataset. Animals in the dataset of this study were directly selected according to their horn phenotype. Therefore, the estimated heritabilities do not represent an estimate for the entire population. Compared to CC and CCL, the BC2 and BC1 binary coding had the lowest and second lowest SNP heritability, respectively. The results of the power analysis (see Additional file 2: Table S3) are in agreement with this finding and demonstrate that the two designs with continuous coded phenotypes are the most appropriate for the detection of the putative QTL involved in the occurrence of scurs. Taking these results and field observations together, it is not correct to consider frontal bumps as smoothly polled as was done in the BC2 coding. Therefore, this coding was not used further in the mapping analysis, and we recommend precise recording of frontal bumps and age of phenotyping for future mapping studies of scurs. The cLDLA of both continuous coded traits CC and CCL gave similar results, with genome-wide significant (BTA5 and BTA12) and suggestive (BTA16, BTA18 and BTA23) signals mapped to the same regions. In contrast, the mapping results of BC1 did not coincide with those of CC and CCL. Both MLMA and cLDLA considered the LOCO approach and variance component estimation and both showed serious convergence problems with BC1 but not with CC and CCL phenotype coding. These convergence problems are most probably the cause for the complete failure of MLMA and the noisy LRT signals of cLDLA for the binary coded scurs trait. In general, this is not the case with binary traits, i.e. MLMA and cLDLA have been successfully applied to fine map and subsequently identify causal mutations for recessive [45] and dominant [18] traits. Moreover, both approaches were also successfully used for highly significant mapping of polygenic traits such as calving ease [46] that resembles CC in coding (five categories) and quantitative nature. Taken together, our results suggest that binary coding, both BC1 and BC2, is an unsuitable oversimplification of a quantitative trait with age-dependent penetrance.

Our well-structured design (one breed, one sex, one polled genotype, one polled allele and recorded age-of-phenotyping) resulted in the mapping of four genome-wide significant loci that affect the development of scurs. The annotation of gene content and subsequent gene set enrichment analysis of the detected regions (see Additional file 2: Table S4) showed that no MGI Mammalian Phenotype level 4 (MMP4) ontology was significantly enriched after correction for multiple testing (adjusted P < 0.05), probably because of the small size of the dataset. Nevertheless, we considered 53 MMP4 with a raw P-value lower than 0.05 to identify putative candidate genes. Among these, we observed 13 ontologies related to bone development, 14 to the blood system, and 7 to the nervous system. Two genes are particularly relevant: *SUCO* and *ARHGAP33*, which account for most of the ontologies associated with bone development and the nervous system, respectively. *SUCO* encodes the SUN domain containing ossification factor (BTA16), which is an essential protein for normal osteoblast function [47]. In mouse, a mutation in this gene causes wide cranial sutures, thin neurocranium, and severe skeletal defects. *ARHGAP33* (BTA18) encodes the neurite outgrowth multiadaptor RhoGAP protein, which is involved in the regulation of dendritic branching during cerebral cortex development [48]. Interestingly, Wang et al. [13] have only very recently highlighted the important role of genes that are involved in nervous system development and in the neural crest cell migration and differentiation, in the differentiation of horn buds.

Taken together, these findings suggest that the scurs phenotype and its genetic background are more complex than previously proposed, and we have severe doubts with a monogenetic inheritance mode. The oversimplified hypothesis about a biallelic *horn* and *scurs* locus is far from the current knowledge about the coordinated action of gene networks during embryogenesis and differentiation of tissues that create organs (e.g. [13]), but this hypothesis still partly guides our mapping designs. One possible explanation would be that the phenotype is caused by a limited number of epistatic effects between the *polled* locus and several, so far unmapped loci that are responsible for scurs, which are part of the accessory genome. Pan-genome analysis would be an option (see [13]) to investigate this hypothesis, but the material and methodology available for our study are not sufficient for such an analysis. In view of our results, we hypothesise that the intensity and course of development of horns and scurs are influenced by several *horn development* genes and environmental factors. It is possible that different variants at the *polled* locus could amplify the transcription of transregulatory RNAs which target genes that are involved in horn development. Thus, these genes would be downregulated or even completely knocked-out. Depending on the degree of downregulation, affected animals may develop scurs, scabs, or bumps or be smoothly polled. The presence of two polled alleles supresses the development of horns and scurs completely. However, if only one polled allele is present, the intensity of this suppression depends on factors such as sex, age, the polled allele and the genetic variance at multiple *horn development* genes, which may be down and upregulated to different degrees depending on some alleles placed at different loci in in the network.

Our investigations demonstrate the complexity of the inheritance of horns. Our results suggest a rather complex network of several interacting genes instead of the previously accepted four loci model. Investigations of the genetic background of peculiar horn phenotypes may provide further insights into the genes that are involved in the development of horns (e.g. [12, 24]). Moreover, the next step to explore the genetic architecture of horn development could be to develop a mapping design in horned animals, e.g. for which the horn length and diameter of not dehorned *pp* animals, which would ideally be already genotyped with an SNP chip used for routine purpose (genomic selection), are measured at comparable ages (e.g. calf markets).

## Conclusions

In this study, we investigated the scurs phenotype in polled Holstein–Friesian cattle and performed a genetic analysis of putative *scurs* loci. We observed a range of horn phenotypes in genetically polled cattle, which almost form a continuous distribution. Our well-structured mapping population led to the identification of four genome-wide significant loci that affect the development of scurs. These results explicitly disagree with the monogenetic inheritance mode that was initially proposed by White and Ibsen [6]. We propose a model in which the complexity of horns, including different horn modifications such as polledness and scurs, and the intensity of horn development are influenced by several genetic factors and non-genetic effects. We assume an oligogenetic architecture and a phenotypically quantitative basis of the trait with age-dependent penetrance.

## Supplementary information

**Additional file 1: Figure S1.** Transformation of the phenotype on a liability scale. Liability (x-axis) with N(0,1). Dashed vertical lines indicate thresholds of each phenotype category according to the respective frequency. 1 = smoothly polled, 2 = frontal bumps, 3 = scabs, 4 = small scurs, 5 = medium scurs. Thresholds: − 0.46, 1.19, 1.81, 2.63; liability values (weighted mean of each section): − 1.111, 0.292, 1.458, 2.110, 2.939.

**Additional file 2: Table S1.** Phenotypic variance explained by all SNPs for all phenotype codings. **Table S2.** Genome-wide significance thresholds for LRT values of cLDLA for different phenotype codings detected by permutation testing. **Table S3.** Power to detect a QTL for the four different codings (Table 1) of 232 high-density genotyped animals. **Table S4.** Gene content of the respective intervals used for the gene set enrichment analysis.

**Additional file 3: Figure S2.** Results of the cLDLA for scurs of BC1 phenotype coding with sw40. LRT-values are shown on the y-axis, bovine chromosomes on the x-axis. The red horizontal line marks the genome-wide significance threshold (α = 0.00005) derived from permutation testing.

**Additional file 4: Table S5.** Positions with significant LRT values of CC and CCL codings.

## Data Availability

The datasets used and analysed during the current study are available from the corresponding author on reasonable request.
