# Supplementary figures and images for: Are scurs in heterozygous polled (Pp) cattle a complex quantitative trait?
Source: Genet Sel Evol. 2020 Feb 7;52:6. doi: 10.1186/s12711-020-0525-z (PMC7006098; doi:10.1186/s12711-020-0525-z)

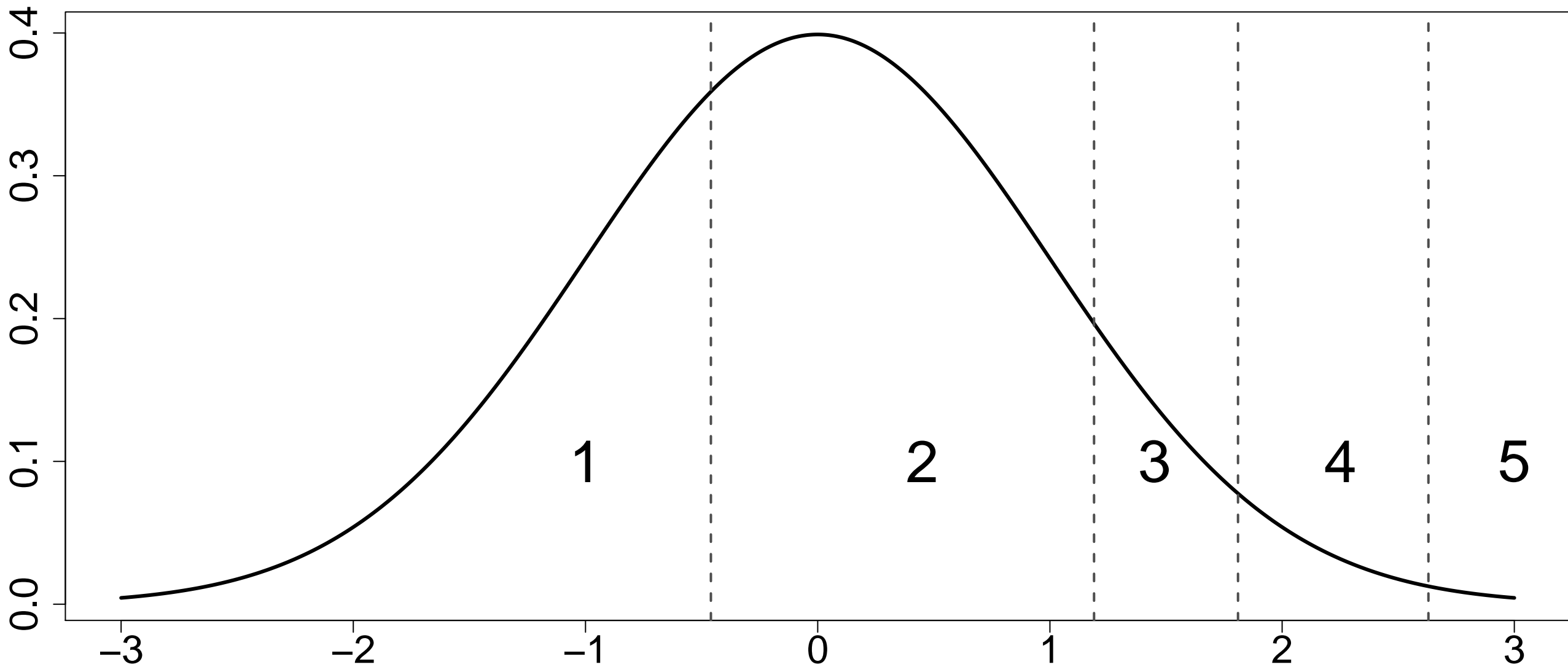

Supplement: Supplementary file 1 — Additional file 1: Figure S1. Transformation of the phenotype on a liability scale. Liability (x-axis) with N(0,1). Dashed vertical lines indicate thresholds of each phenotype category according to the respective frequency. 1 = smoothly polled, 2 = frontal bumps, 3 = scabs, 4 = small scurs, 5 = medium scurs. Thresholds: − 0.46, 1.19, 1.81, 2.63; liability values (weighted mean of each section): − 1.111, 0.292, 1.458, 2.110, 2.939. [file 12711_2020_525_MOESM1_ESM.pdf]

# Coding BC1

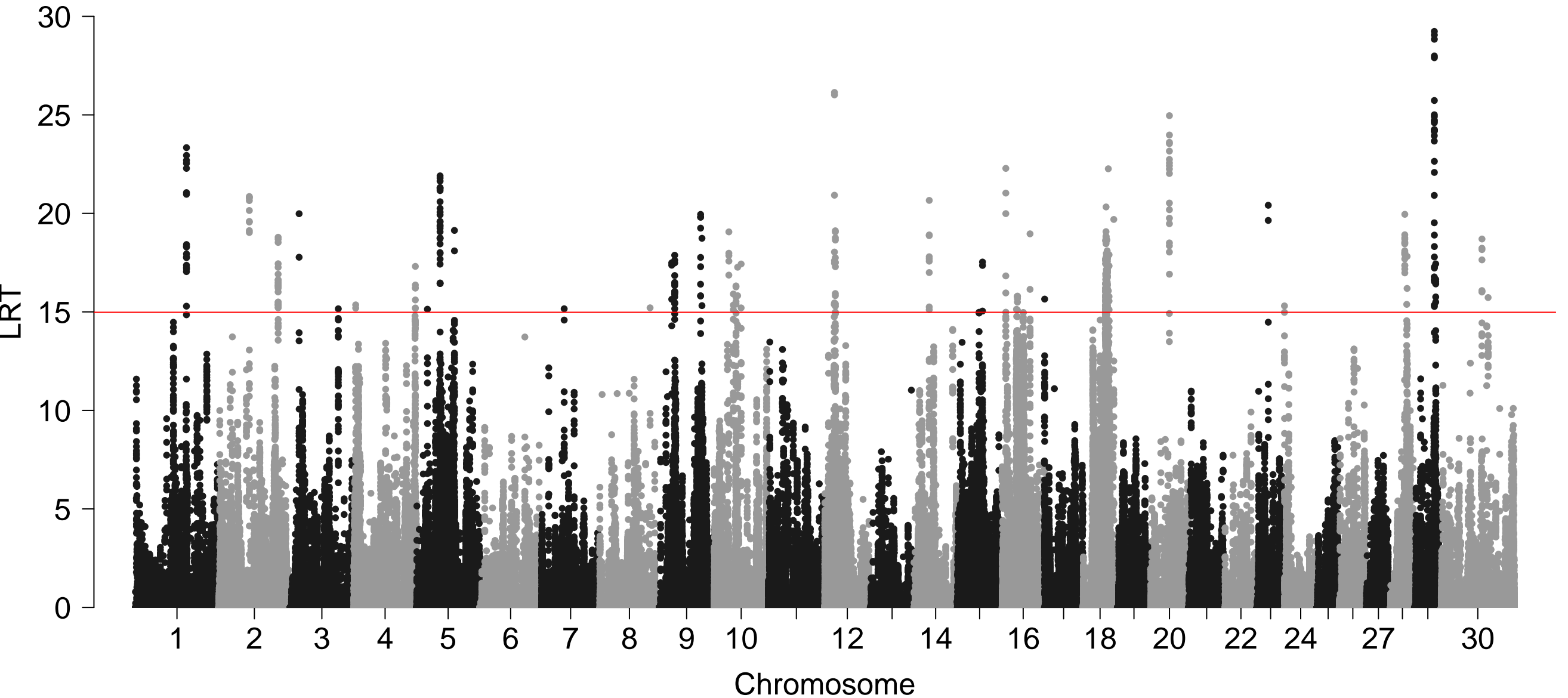

Supplement: Supplementary file 3 — Additional file 3: Figure S2. Results of the cLDLA for scurs of BC1 phenotype coding with sw40. LRT-values are shown on the y-axis, bovine chromosomes on the x-axis. The red horizontal line marks the genome-wide significance threshold (α = 0.00005) derived from permutation testing. [file 12711_2020_525_MOESM3_ESM.pdf]
