# Supplementary material for: Are scurs in heterozygous polled (Pp) cattle a complex quantitative trait?
Source: Genet Sel Evol. 2020 Feb 7;52:6. doi: 10.1186/s12711-020-0525-z (PMC7006098; doi:10.1186/s12711-020-0525-z)
Supplement: Supplementary file 2 — Additional file 2: Table S1. Phenotypic variance explained by all SNPs for all phenotype codings. Table S2. Genome-wide significance thresholds for LRT values of cLDLA for different phenotype codings detected by permutation testing. Table S3. Power to detect a QTL for the four different codings (Table 1) of 232 high-density genotyped animals. Table S4. Gene content of the respective intervals used for the gene set enrichment analysis. [file 12711_2020_525_MOESM2_ESM.docx]

**Table S1 Phenotypic variance explained by all SNPs for all phenotype codings**

| **Coding** | **Dataset** |  | **V(G)** | **V(e)** | **Vp** |  | **V(G)/Vp** |
| --- | --- | --- | --- | --- | --- | --- | --- |
| **CC** | **Observed phenotype** | **Estimate** | 0.2889 | 0.1577 | 0.4466 |  | 0.6469 |
|  |  | **SE** | 0.0998 | 0.0812 | 0.0505 |  | 0.1900 |
|  | **Mean permuted** | **Estimate** | 0.0551 | 0.4803 | 0.5355 |  | 0.1036 |
|  |  | **SE** | 0.1145 | 0.1186 | 0.0583 |  | 0.2106 |
| **CCL** | **Observed phenotype** | **Estimate** | 0.4168 | 0.2504 | 0.6672 |  | 0.6247 |
|  |  | **SE** | 0.1543 | 0.1272 | 0.0756 |  | 0.1995 |
|  | **Mean permuted** | **Estimate** | 0.0588 | 0.7242 | 0.7830 |  | 0.0741 |
|  |  | **SE** | 0.1666 | 0.1738 | 0.0851 |  | 0.2104 |
| **BC1** | **Observed phenotype** | **Estimate** | 0.1139 | 0.0754 | 0.1894 |  | 0.6017 |
|  |  | **SE** | 0.0538 | 0.0451 | 0.0219 |  | 0.2513 |
|  | **Mean permuted** | **Estimate** | 0.0179 | 0.2009 | 0.2188 |  | 0.0806 |
|  |  | **SE** | 0.0465 | 0.0485 | 0.0238 |  | 0.2102 |
| **BC2** | **Observed phenotype** | **Estimate** | 0.0329 | 0.0474 | 0.0803 |  | 0.4100 |
|  |  | **SE** | 0.0154 | 0.0146 | 0.0088 |  | 0.1776 |
|  | **Mean permuted** | **Estimate** | 0.0106 | 0.0923 | 0.1030 |  | 0.1022 |
|  |  | **SE** | 0.0220 | 0.0228 | 0.0112 |  | 0.2101 |

**Table S2 Genome-wide significance thresholds for LRT values of cLDLA for different phenotype codings detected by permutation testing**

| sw | CC coding | CCL coding | BC1 coding |
| --- | --- | --- | --- |
| 20 | 15.40 |  |  |
| 40 | 14.74 | 15.28 | 14.98 |
| 80 | 16.39 |  |  |
| 160 | 15.67 |  |  |

sw: sliding window

**Table S3 Power to detect a QTL for the four different codings (Table 1) of 232 high-density genotyped animals**

| Coding | Significance level | Genetic variance explained by QTL | | |
| --- | --- | --- | --- | --- |
|  |  | 10% | 30% | 50% |
| CC | Genome wide | 0.0074 | 0.4769 | 0.9627 |
|  | Suggestive | 0.0327 | 0.7055 | 0.9918 |
| CCL | Genome wide | 0.0078 | 0.5088 | 0.9713 |
|  | Suggestive | 0.0321 | 0.7299 | 0.9939 |
| BC1 | Genome wide | 0.0023 | 0.2370 | 0.8145 |
|  | Suggestive | 0.0127 | 0.4478 | 0.9356 |
| BC2 | Genome wide | 0.0003 | 0.0261 | 0.2025 |
|  | Suggestive | 0.0026 | 0.0962 | 0.4275 |

**Table S4 Gene content of the respective intervals used for the gene set enrichment analysis**

| BTA | Start (bp) | End (bp) | Gene content |
| --- | --- | --- | --- |
| 05 | 44,553,747 | 44,795,065 | CPSF6 |
| 12 | 7,274,577 | 8,344,252 | -- |
| 12 | 18,056,088 | 19,109,141 | FNDC3A* |
| 12 | 20,354,904 | 21,292,686 | RNASEH2B, GUCY1B2, SNRNP35, C13orf42, FAM124A, SERPINE3, INTS6, WDFY2, DHRS12 |
| 16 | 39,971,282 | 41,008,908 | SUCO, FASLG, NPM1, TNFSF18, TNFSF4, RPL7 |
| 18 | 46,291,711 | 46,491,711 | KMT2B, PROSER3, ARHGAP33, RBM42, ETV2, COX6B1, UPK1A, ZBTB32, IGFLR1, U2AF1L4, PSENEN, LIN37, HSPB6, HAUS5 |
| 23 | 46,227,175 | 47,272,519 | SLC35B3* |

BTA: Bos taurus autosome; Start and end position in bp of the 200kbp (1Mbp) surrounding interval of the detected chromosome region; gene content of the interval; *200kpb interval was devoid of genes, closest gene in 5’3’ orientation within a 1Mbp surrounding interval was used.
